# Effects of incomplete inter-hospital network data on the assessment of transmission dynamics of hospital-acquired infections

**DOI:** 10.1371/journal.pcbi.1008941

**Published:** 2021-05-06

**Authors:** Hanjue Xia, Johannes Horn, Monika J. Piotrowska, Konrad Sakowski, André Karch, Hannan Tahir, Mirjam Kretzschmar, Rafael Mikolajczyk

**Affiliations:** 1 Institute for Medical Epidemiology, Biometrics and Informatics (IMEBI), Interdisciplinary Center for Health Sciences, Medical School of the Martin-Luther University Halle-Wittenberg, Halle, Saxony-Anhalt, Germany; 2 Institute of Applied Mathematics and Mechanics, University of Warsaw, Warsaw, Poland; 3 Institute of High Pressure Physics, Polish Academy of Sciences, Warsaw, Poland; 4 Institute for Epidemiology and Social Medicine, University of Münster, Münster, North Rhine-Westphalia, Germany; 5 Julius Center for Health Sciences & Primary Care, University Medical Center Utrecht, Utrecht University, Utrecht, The Netherlands; Institute for Disease Modeling, UNITED STATES

## Abstract

In the year 2020, there were 105 different statutory insurance companies in Germany with heterogeneous regional coverage. Obtaining data from all insurance companies is challenging, so that it is likely that projects will have to rely on data not covering the whole population. Consequently, the study of epidemic spread in hospital referral networks using data-driven models may be biased. We studied this bias using data from three German regional insurance companies covering four federal states: AOK (historically “general local health insurance company”, but currently only the abbreviation is used) Lower Saxony (in Federal State of Lower Saxony), AOK Bavaria (in Bavaria), and AOK PLUS (in Thuringia and Saxony). To understand how incomplete data influence network characteristics and related epidemic simulations, we created sampled datasets by randomly dropping a proportion of patients from the full datasets and replacing them with random copies of the remaining patients to obtain scale-up datasets to the original size. For the sampled and scale-up datasets, we calculated several commonly used network measures, and compared them to those derived from the original data. We found that the network measures (degree, strength and closeness) were rather sensitive to incompleteness. Infection prevalence as an outcome from the applied susceptible-infectious-susceptible (SIS) model was fairly robust against incompleteness. At incompleteness levels as high as 90% of the original datasets the prevalence estimation bias was below 5% in scale-up datasets. Consequently, a coverage as low as 10% of the local population of the federal state population was sufficient to maintain the relative bias in prevalence below 10% for a wide range of transmission parameters as encountered in clinical settings. Our findings are reassuring that despite incomplete coverage of the population, German health insurance data can be used to study effects of patient traffic between institutions on the spread of pathogens within healthcare networks.

## Author summary

Patterns of patients’ transfer between different hospitals contribute crucially to the risk of hospital-acquired infections (HAIs) in the health care system. To quantify this risk, network models can be applied. The estimated risk can be inaccurate in the case of incomplete data on hospital admissions, which can be a consequence of the multiplicity of insurance companies as it is the case in Germany. To develop a better understanding of how incompleteness of data affects network measures and the simulated spread of HAI, we compared those measures derived from sampled, scale-up and original data, based on hospitalization data from three AOK insurance companies. We found that common network measures were affected by incompleteness, but the simulated prevalence as a measure of epidemic spread in the network was robust over a large range of incompleteness proportions. Epidemics and the transition of the infectious diseases may be modelled on hospital data with a coverage as low as 10% of the local population, whilst maintaining accuracy to within 10% of the true population prevalence.

## Introduction

Transfers of patients between hospitals have an important impact on transmission pathways of hospital-acquired infections (HAIs) [1–11]. In recent years, hospital discharge databases based on English, Dutch as well as French national medical registration datasets have been used to construct “healthcare networks” to provide insights into patient transfer management, hospital infection prevention and control [2, 3, 5, 7, 8, 11]. In these networks, nodes represent hospitals and edges between pairs of nodes represent patient transfers between the linked pairs of hospitals. Based on these data, network measures like degree, closeness, and also network density were calculated [4, 5, 8]. The networks were used for simulating the spread of HAIs, evaluating epidemic risk [1–3, 5–7, 9–11], and recommending control strategies [1, 5, 11, 12].

In Germany, a central discharge database does not exist. In 2020, there were 105 statutory and several private health insurance companies in Germany. More than 90% of the population are insured by any one of the statutory health insurance companies and every insurant can freely choose any of these at any time, so that changes between companies occur frequently. It is very challenging to obtain data from all insurance companies, so that any projects based on empirical data will suffer from incomplete data access. Statistical properties of the network and the modelling predictions based on these incomplete data can be biased. The incomplete data may for example lead to missing edges in a network graph representing hospital connections. For person-to-person contact networks, incompleteness is a common problem; thus, several studies have focused on inferring network statistics from incomplete contact data in various contexts [13–22]. Nevertheless, the effects of incomplete patient transfer data have not been studied previously.

Thus, in this paper we aimed to assess how different levels of data incompleteness affect estimates of network measures and modelled infection spread.

## Materials and methods

### Data processing

We used anonymized data from hospitalization databases, provided by three different German insurance companies, specific to each region: AOK (historically “general local health insurance company”, but currently only the abbreviation is used) Lower Saxony, AOK Bavaria, and AOK PLUS (merger of AOK Saxony and AOK Thuringia). AOKs are insurance companies, which historically exclusively insured persons from the federal states where they were founded. As a consequence, they have high coverage of the population in their own federal state and low coverage outside, and can thus be used to study regional networks. In each dataset, the following are available: anonymized patient ID, anonymized hospital facility ID, the federal state the hospital is located in, admission and discharge date, main diagnosis (ICD 10 GM code) as well as year of birth and sex of the patient.

**Fig 1:**
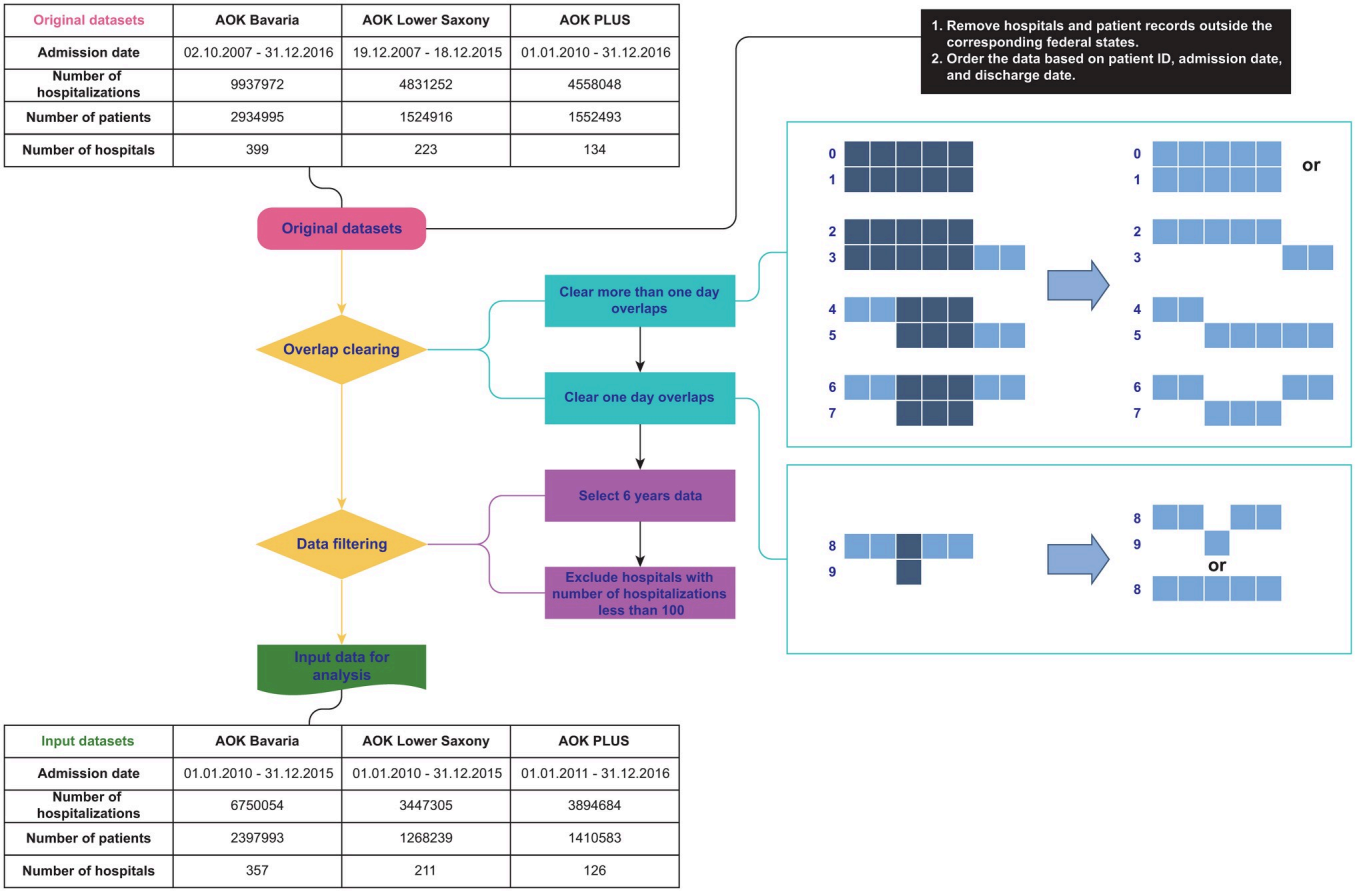
Data filtering process.

The three datasets covered different time spans. To ensure comparability, we studied data from each across the interval of same length of six-years. Some hospitals were not active during the whole time and based on observed distribution of hospitalizations, we excluded hospitals with less than 100 hospitalizations during this six-year time period from further analysis (see S1 Fig). We also excluded all hospitals located outside the respective federal state as they did not have the same local coverage of the studied population in their catchment area. The datasets contained various types of overlaps, which suggested that a patient stayed in multiple hospitals on a given day [23, 24]. Overlaps can happen due to several factors. If, for example, a hospitalization is completely included in another hospitalization at a different hospital, the patient may have been moved from one hospital to another before returning to the originating facility. In this case, for reimbursement purposes, the second stay had to be considered as part of the first, resulting in the record of continuous hospitalization in the first hospital. However, not all overlaps could be explained following this logic. If patients appeared in multiple hospitals simultaneously at a one-day overlap (indicating possibly a coding error for a direct transfer), we randomly chose one of hospitals. The filtering procedures are in detail in Fig 1.

### Surrogate data construction

#### Construction of sampled and scale-up data

We considered the original data provided by the companies as a starting point (Table 1). By randomly removing a fraction of patients from these datasets, we obtained a subset with remaining patients, defined as sampled dataset.

**Table 1:** Description of health insurance datasets.

Dataset	Time span	Population coverage*	Hospitalization coverage*
AOK Bavaria	2010–2015	33.9%	38.3%
AOK Lower Saxony	2010–2015	30.7%	34.2%
AOK PLUS	2011–2016	44.0%	40.3%

* Percentage of population and all hospitalizations in the federal state based on the year 2015 [25, 26].

In the next step, we randomly resampled patients from the sampled datasets to compensate for the excluded patients. For each resampling, once the patient was chosen, their information was cloned and labelled as a new patient. The progress was repeated until there was the same number of patients in the resampled dataset as there was in the original dataset.

### Network construction

Two distinct types of networks were defined to represent the patient transfer data. One type was a time-independent (static) network, defined as a hospital network as by Iwashyna et al. [27], where patient transfers between the hospitals within a certain period were described. This network had *N* nodes and *e* directed weighted edges. In this network, nodes represented the hospitals and edges starting from node *i* towards node *j* with weight indicating the total number of patient transfers from hospital *i* to hospital *j* during the whole time period [28]. From this static network, we further extracted two different kinds of subnetworks: The first one consisted only of patient transfers from one hospital to another with no stay at home in between; we referred to this as the “direct hospital network”. The other network consisted solely of indirect transfers, i.e. transfers where patients stayed at home for at least one day between discharge from one hospital and admission to the next one. The resulting transfer network, we termed the “indirect hospital network”.

The second type of network was a time-dependent (transient) two-mode network [29], named patient-movement network (also known as affiliation or bipartite network). In this approach, there were two distinct sets of nodes: one was the set of all individuals, and the other one was the set of all hospitals (as well as one community node for individuals currently not hospitalized). In this type of network, links existed only between nodes belonging to different sets. This network was composed of *M* patient nodes and *N* + 1 location nodes. All nodes in this network were preserved during the covered period.

In our study, we used the static network for calculating the network measures, and the transient network for running the epidemic model, which obeyed the actual patient locations.

### Description of network measures and epidemic models

Since network measures are widely utilized for describing the importance of hospitals in hospital networks, we adopted some commonly used ones for describing hospital network structures.

#### Hospital network measures

Each static weighted directed network *G*(*E*, *V*) with *E* the edge set and *V* the vertex set can be represented by a *N* × *N* adjacency matrix *A*, defined as

$$\begin{array}{c} A_{ij}=\{\begin{array}{cc} w_{ij}, & \text{if}\;\{i,j\}\in E\;\text{and}\;i\neq j\text{;}\\ 0, & \text{otherwise.} \end{array} \end{array}$$ (1)

Here, network weight *w*_*ij*_ denotes the total number of patient transfers during 6 years from node *i* to *j* and *N* is the number of hospitals. To depict the network cohesion, we calculated the network density, defined as $\rho =\frac{e}{N^{2}}$ where *e* denotes the number of network edges. Moreover, we considered four additional network measures with further subdivision regarding to directions: in- and out-degree, in- and out-strength, the shortest path, as well as in- and out-closeness.

Degree and strength were used for describing the transfer activity of a node. For node *i*, its in- and out-degrees are defined by $k_{i}^{in}=\sum_{j=1}^{N}sgn\left(w_{ji}\right)$ and $k_{i}^{out}=\sum_{j=1}^{N}sgn\left(w_{ij}\right)$, respectively, which indicate the number of neighbour hospitals of hospital *i*. Analogously, its in- and out- strengths are given by $s_{i}^{in}=\sum_{j=1}^{N}w_{ji}$ and $s_{i}^{out}=\sum_{j=1}^{N}w_{ij}$, which stand for the number of ingoing and outgoing patients of hospital *i*, respectively. *sgn* is the sign function with

$$\begin{array}{c} sgn\left(x\right)=\{\begin{array}{cc} -1 & \text{,}\;\text{if}\;x<0\text{;}\\ 0 & \text{,}\;\text{if}\;x=0\text{;}\\ 1 & \text{,}\;\text{if}\;x>0\text{.} \end{array} \end{array}$$ (2)

With respect to pathogen propagation, it was essential to determine the distances or shortest paths between hospitals [8]. The shortest path through the static weighted directed network from node *i* to node *j* is defined as $d_{ij}=\min_{h}(\frac{1}{w_{ih}}+\cdots +\frac{1}{w_{hj}})$, *h* ∈ *V*\{*i*, *j*}. It was used for calculating the measure “closeness” [8, 30].

In addition, we calculated the in- and out-closeness centralities of node *i*: $C_{i}^{in}=\sum_{j=1}^{N}\frac{1}{d_{ji}}$ also $C_{i}^{out}=\sum_{j=1}^{N}\frac{1}{d_{ij}}$ [8, 30]. The closeness determines the risk posed by an outbreak in any of the other hospitals [8]. In-closeness $C_{i}^{in}$ indicates that the outbreak risk in hospital *i* was triggered by receiving patients from other hospitals. Out-closeness $C_{i}^{out}$ shows the risk caused by hospital *i* in its neighbour hospitals.

#### Patient-movement based epidemic models

We used a susceptible-infectious-susceptible (SIS) model and applied parameters for hospital-acquired methicillin-resistant Staphylococcus aureus (MRSA). Patients colonized or infected with MRSA were defined as “Infectious”. In the following, we defined “become infected” not exclusively as infection, but also as colonization by the pathogen. On modelling day *t*, we first identified the location of every patient based on the transient network. Then, by keeping their epidemic statuses on day *t* − 1, we calculated the total number of patients $N_{L}^{t}$ as well as the number of infectious patients $N_{L,I}^{t}$ in location *L*. Finally, we ran the following epidemic models and calculated the prevalence of infection in hospitals and the community node, defined as the proportion of infectious patients according to the patient status on day *t*.

In our model, a susceptible patient became infected with probability $\beta_{L}^{t}$ on day *t* through contacts with infectious patients at the same location *L* on day *t*. The probability of becoming infected at location *L* on day *t* was

$$\begin{array}{c} \beta_{L}^{t}=\{\begin{array}{cc} \frac{N_{L,I}^{t}}{N_{L}^{t}-1}\cdot \beta , & N_{L,I}^{t}\geq 1\;\text{and}\;N_{L}^{t}\geq 2\text{;}\\ 0, & \text{otherwise.} \end{array} \end{array}$$ (3)

Here, $N_{L,I}^{t}$ denoted the number of infectious patients at location *L* on day *t* and $N_{L}^{t}$ was the total number of patients with *t* = *T*_1_, *T*_1_ + 1, ⋯, *T*_2_. *T*_1_ and *T*_2_ were the start and end days of the simulation. Here, we assumed that the population mixed homogeneously within each location.

For the initial state of every simulation, we randomly selected 4% of the patients to be infectious [31–33]. We assumed that no transmission occurred in the community node, and that the discharged patients remained infectious until they recovered. Infectious patients recovered spontaneously with probability *γ* per day. Following Scanvic et al. [34] and Donker et al. [11], we set $\gamma =\frac{1}{365}{days}^{-1}$ based on the assumption that the mean time of MRSA colonization was 365 days. We assumed that the recovery probabilities in different hospitals and the community were identical. Following the previous work of Donker et al. [11], we assumed the reference transmission probability per patient contact to be *β* = 0.085 in hospital nodes and *β* = 0 in the community node. Since we used stochastic approach to simulate pathogen spread within the network, we performed 100 independent simulations for each set of values (e.g. different levels of data incompleteness).

#### Measures used for comparison of network characteristics and spread of pathogens

- **Absolute value of relative bias (ARB)**: As different network measures, applied on the hospital network, yielded deviations in varying degrees, we computed the ARB for sampled and scale-up datasets in comparison with the original data at node *i*:
  $$\begin{array}{c} {{ARB}_{M}({Net}_{O},{Net}_{E})}_{i}=|\frac{M_{i}^{\left({Net}_{O}\right)}-M_{i}^{\left({Net}_{E}\right)}}{M_{i}^{\left({Net}_{O}\right)}}|, \end{array}$$
  where *M*_*i*_ denoted the weighted directed static network measure (degree, strength, or Closeness) at node *i*. *Net*_*O*_ and *Net*_*E*_ represented the network based on the original dataset and sampled or scale-up dataset, respectively. As the nodal ARB did not take into account that different nodes can correspond to very different number of patient hospitalizations and transfers, we also considered the weighted ARB, defined as:
  $$\begin{array}{c} {ARB}_{M}^{w}({Net}_{O},{Net}_{E})=\sum_{i=1}^{N}H_{i}^{W}\cdot {{ARB}_{M}({Net}_{O},{Net}_{E})}_{i}\;, \end{array}$$
  where $H_{i}^{W}=\frac{H_{i}\left(Net_{O}\right)}{\sum_{j=1}^{N}H_{j}\left(Net_{O}\right)}$, *H*_*i*_ was the total number of patient admissions at node i and *N* was the number of hospitals.
  The values for ARB and weighted ARB ranged from 0 to 1. Values close to 0 indicated that the estimation based on the surrogate data was close to the one based on the original data.
- **Cosine similarity (CS)**: We further applied CS as a similarity measure defined for vectors. The value of CS was bounded between −1 and + 1, with a value of 1 for parallel vectors with the same orientation, a value of −1 for parallel vectors with opposite orientation, and 0 for perpendicular vectors [35]. The local CSs of node *i* were defined as:
  $$\begin{array}{c} CS^{in}{\left(Net_{O},Net_{E}\right)}_{i}=\frac{\sum_{j}w_{ji}^{Net_{O}}\cdot w_{ji}^{Net_{E}}}{\sqrt{\sum_{j}{\left(w_{ji}^{Net_{O}}\right)}^{2}}\sqrt{\sum_{j}{\left(w_{ji}^{Net_{E}}\right)}^{2}}} \end{array}$$ (4)
  $$\begin{array}{c} CS^{out}{\left(Net_{O},Net_{E}\right)}_{i}=\frac{\sum_{j}w_{ij}^{Net_{O}}\cdot w_{ij}^{Net_{E}}}{\sqrt{\sum_{j}{\left(w_{ij}^{Net_{O}}\right)}^{2}}\sqrt{\sum_{j}{\left(w_{ij}^{Net_{E}}\right)}^{2}}} \end{array}$$ (5)
  $w_{ij}^{Net_{O}}$ and $w_{ij}^{Net_{E}}$ were the numbers of total patient movements from hospital *i* to its neighbours *j* in *Net*_*O*_ and *Net*_*E*_ during considered years, respectively. In addition, to study the global similarity, we calculated the weighted CSs:
  $$\begin{array}{c} CS^{W,in}\left(Net_{O},Net_{E}\right)=\sum_{i=1}^{N}H_{i}^{W}\cdot CS^{in}{\left(Net_{O},Net_{E}\right)}_{i} \end{array}$$ (6)
  $$\begin{array}{c} CS^{W,out}\left(Net_{O},Net_{E}\right)=\sum_{i=1}^{N}H_{i}^{W}\cdot CS^{in}{\left(Net_{O},Net_{E}\right)}_{i} \end{array}$$ (7)
  $H_{i}^{W}$ was defined as in the above section on ARB.
- **Prevalence relative bias (PRB) and final PRB**: To evaluate the estimation of daily prevalence in the SIS model, we then computed PRB as follow:
  $$\begin{array}{c} PRB{\left(Net_{O},Net_{E}\right)}_{t}=\frac{Pr_{t}^{Net_{O}}-Pr_{t}^{Net_{E}}}{Pr_{t}^{Net_{O}}} \end{array}$$ (8)
  *Pr*_*t*_ was the prevalence on day *t* with *t* = *T*_1_, *T*_1_ + 1, ⋯, *T*_2_, which had been defined in the section “Patient-movement based epidemic models”. The time-dependent PRB reflected the underestimated proportion of the daily prevalence if *PRB* > 0, while *PRB* < 0 indicated the overestimated proportion. Since PRB depended on time, we additionally defined a measure “final PRB” by averaging the PRB values in their steady state.

#### Threshold for preservation of transmission networks regarding incompleteness

Subsequently, we investigated at which threshold of incompleteness the network characteristics were still reasonably preserved and estimates of epidemic spread are maintained. We considered error levels of 5% and 10%, measured by final PRB as acceptable. In addition to the original transmission parameters, we studied the error levels for varying parameters for *β* and *γ* in the patient-movement based epidemic models.

#### Software

R package “tnet” [36] was used for creating and analysing the hospital networks. Own code was written for the stochastic SIS simulation. To visualize the data we used the R package “ggplot2” [37]. For all analyses, we used R version 4.0.3 [38].

## Results

### Description of the AOK networks

The data from AOK PLUS included more than 40%, whereas AOK Bavaria as well as AOK Lower Saxony included around 30% of the population of the corresponding federal states (Table 1 in section Materials and Methods).

The hospital network and patient characteristics of the original AOK datasets were presented in Tables 2 and 3. Based on the network densities *ρ*^*DT*^ and *ρ*^*in*−*DT*^ (see Table 2), we inferred that the indirect hospital networks had much more connections than the direct networks. The average number of hospitalizations and average length of stay (LOS) from different AOK datasets were close to each other, whereas the average LOS in AOK PLUS was substantially higher than in the other two datasets (Table 3).

**Table 2:** Basic description of AOK hospital networks.

Dataset	No. of hospitals (*N*)	*ρ*^*DT*^	*ρ*^*in*−*DT*^	${\bar{w}}^{DT}$	${\bar{w}}^{in-DT}$
AOK Bavaria	357	0.10	0.37	3.29	30.86
AOK Lower Saxony	211	0.13	0.41	4.47	44.47
AOK PLUS	126	0.28	0.61	12.25	144.23

The static networks had *N* nodes; *ρ* represented the network density with the subscripts “DT” and “in-DT” denoting the direct and indirect hospital networks. The “$\bar{w}$” columns gave the average number of patient transfers per day between different hospitals.

**Table 3:** Basic description of AOK patient characteristics.

Dataset	No. of patients (*M*)	Avg. No. of hospitalizations per person	Avg. No. of persons per day per hospital	Avg. LOS
AOK Bavaria	2,397,993	2.81	76.80	8.90
AOK Lower Saxony	1,268,239	2.72	66.23	8.88
AOK PLUS	1,410,588	2.76	136.74	9.70

The transient network included *M* individuals who were insured by the AOKs and were hospitalized at least once during the 6 years period. The abbreviation “Avg.” was short for “average”, “No.” for “number”, and “LOS” for the “length of stay”.

To provide further information about the data, we showed also the distribution of LOS in S2–S4 Figs, of hospitalizations in S5 Fig, of network weights in S6 Fig, of in- and out-degrees of each hospital in S7 and S8 Figs, and of in- and out-strengths in S9 and S10 Figs. More detailed information about the AOK data can be also accessed in our previous work [23, 24].

### Effects of incompleteness on estimated network characteristics

**Fig 2:**
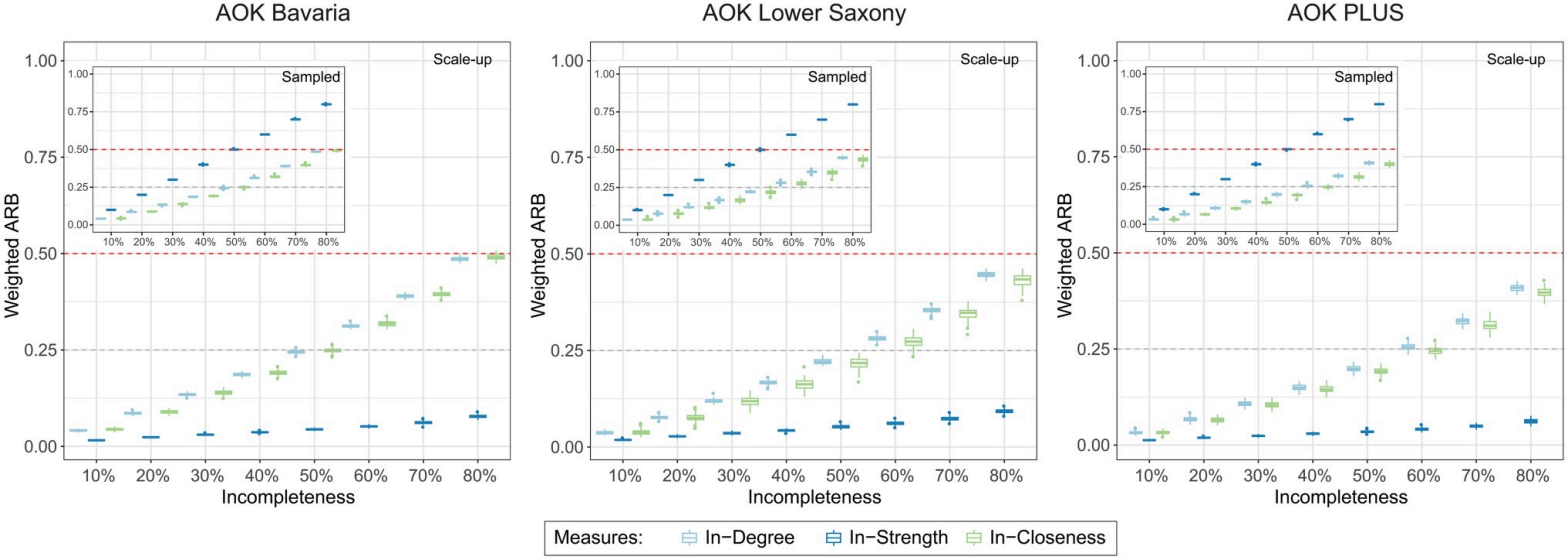
Impact of sampling and scale-up on direct hospital network measures (visualized in boxplots). The weighted absolute value of relative biases (ARBs) across different network measures on sampled (inset) and scale-up (main plot) datasets for various incompleteness levels. Incompleteness was defined as the percentage of removed patients from the original datasets.

The static network measures: in- and out-degree, in- and out-strength, and in- and out-closeness were calculated for the sampled and scale-up datasets. The results were compared with the ones evolving from the original datasets by using the evaluation measure ARB (Fig 2).

Despite the fact that there were differences in basic network properties in the various insurance datasets, the ARBs of different network measures at different incompleteness levels were similar across these datasets (see also Supporting information S11 Fig). There was also no strong difference in ARBs between direct and indirect networks in each dataset. Among all network measures, only strength showed a difference between sampled and scale-up datasets.

To further assess the impacts of incompleteness on network characteristics, we used cosine similarity (CS). The findings are presented in S12–S15 Figs. The CS showed more stable results in the dataset of the AOK PLUS, which had less area of low CS values at the same incompleteness level as the other two datasets. When incompleteness reached 90%, several middle and small hospitals displayed CS values lower than 0.6 and 0.7 in AOK Bavaria and AOK Lower Saxony, while there were very few low CS values in AOK PLUS and only in some small hospitals. The indirect hospital networks were less affected by incompleteness than the direct hospitals. The scale-up of the sampled dataset to the original size provided little improvement.

**Fig 3:**
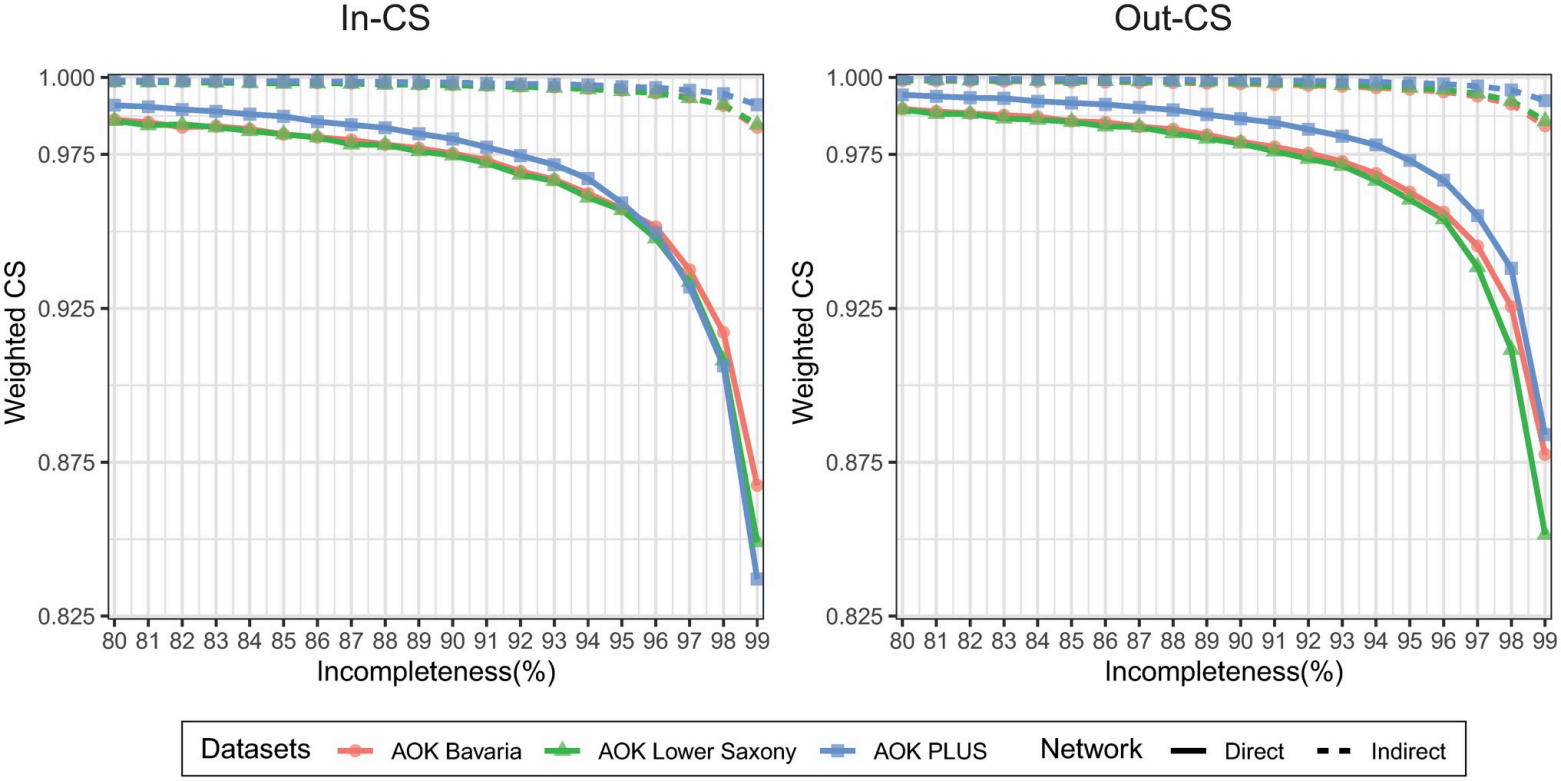
Weighted cosine similarities (CSs). Weighted CSs in directions “in” and “out” between the sampled networks and the whole hospital networks as a function of the incompleteness levels for three insurance datasets. The solid and dashed lines represented the weighted CSs on direct and indirect hospital networks based on these AOK datasets, respectively.

To investigate the overall effect of incompleteness on patient transfer patterns, we calculated the weighted CS. Direct networks were affected by lower degrees of incompleteness than indirect networks, which displayed CS above 0.98 up to incompleteness of 97–98% (Fig 3).

### Effects of incompleteness on simulated spread of infections

**Fig 4:**
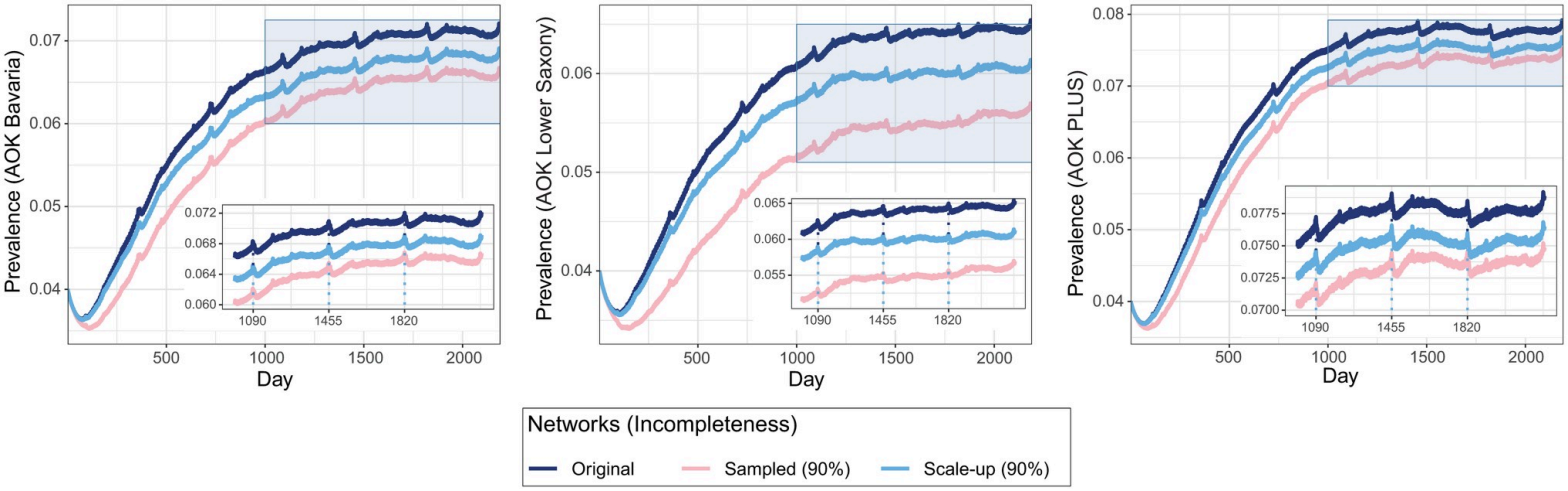
Prevalence in community nodes obtained from transmission model simulations for different AOK datasets. The dashed lines in each graph point the x-axis of prevalence peaks.

The daily prevalence was hardly affected by incompleteness as high as 90% of the original datasets, when scale-up was applied (S16–S18 Figs). Even at higher incompleteness levels the scale-up of the data markedly reduced the bias in estimation of prevalence, which slightly decreased for less complete data (see S16–S18 Figs). There were fluctuations in prevalence related to holidays and weekends when there were fewer patients in the hospitals (see Fig 4 and S19 Fig). This was especially true for the time between Christmas and New Year. These seasonal patterns were consistent across years and AOK datasets. Overall, incompleteness had less impact on prevalence in hospitals than on the prevalence in the community node.

### Incompleteness threshold for preservation of transmission characteristics

**Fig 5:**
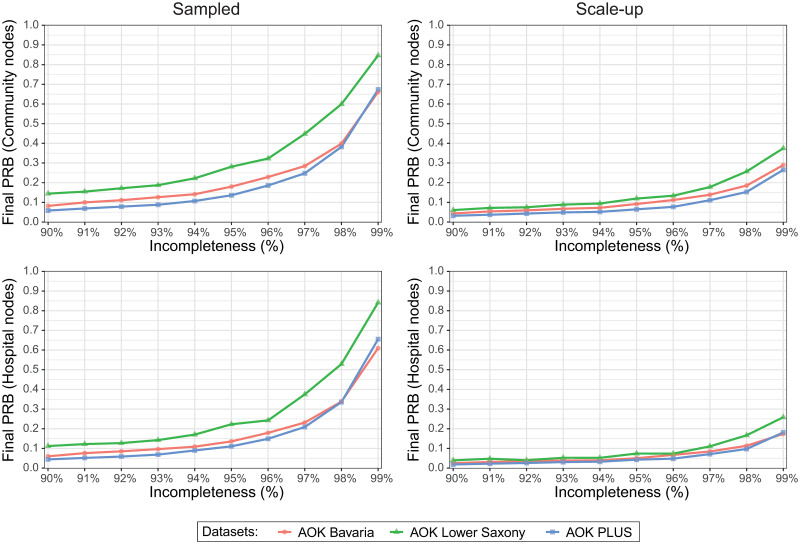
Final PRBs of simulated networks for each AOK plotted against percentage of incompleteness.

After determining that the effects of incompleteness became visible, when incompleteness levels surpassed 90%, we further focussed on this area and studied the higher incompleteness more in detail (Fig 5). At this high incompleteness level, the scale-up datasets performed better than sampled datasets. In the sampled datasets, AOK Lower Saxony appeared less robust to higher incompleteness levels compared to the other two datasets. 10% PRB was reached already at 90% incompleteness in the AOK Lower Saxony and at 94–95% incompleteness in the AOK Bavaria and AOK PLUS datasets (for the community node prevalence). When additional, scale-up is applied, even higher incompleteness levels can be tolerated.

**Fig 6:**
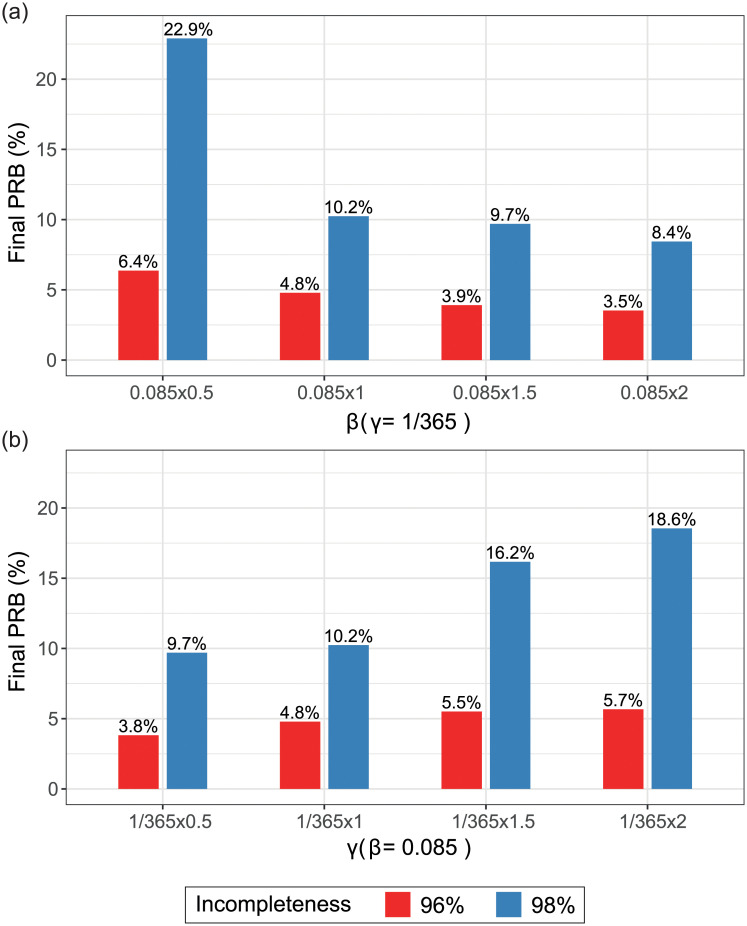
Final prevalence relative biases (PRBs) in hospital nodes for varying transmission parameters. The final PRBs in hospital nodes were calculated based on the simulations on AOK PLUS patient-movement networks, built by scale-up at incompleteness levels of 96% and 98%. In (a) we presented the final PRBs in hospital nodes for different values of *β*. In (b) we presented the final PRBs in hospital nodes for different values of *γ*.

To further investigate the effects of incompleteness on the final PRBs based on the SIS model, we used the AOK PLUS data with varying *β* and *γ* values. We could show that even across a wide range of values (reflecting various pathogen with realistic characteristics) final PRBs were preserved for high incompleteness levels of 96% (see Fig 6 and S20 Fig). For even higher incompleteness levels, there was some indication that for some combination of parameters final PRBs became substantially larger.

## Discussion

We demonstrated that while incomplete coverage of the population affected the studied network measures, this did not greatly bias the prevalence as a measure of epidemic spread until the level of incompleteness exceeded 90% (in relation to the original dataset). Scale-up by “cloning” the patients provided little improvement, unless for very high incompleteness levels.

Previous research demonstrated that the epidemic risk of single hospitals in the absence of transmission models can be estimated by utilizing their network measures [31]. Unfortunately, incomplete data would affect such estimates.

Degree and closeness displayed strong bias of around 25% at incompleteness levels of 50%–60% and nearly 50% at an incompleteness level of 80%. This effect was mainly due to the removal of weaker links, caused by missing patient records with transfers between particular hospitals, which would underestimate the risk of pathogen transmission between those hospitals. In case of the network measure strength, this bias was reduced to 10% or less by adopting the scale-up method based on “cloning” of patients. However, since the scale-up could not impute lost edges, it provided little benefit with respect to the other measures.

The CS as measures of affinity was relatively high even at high incompleteness levels, likely due to the fact that removal of patients occurred at random, as demonstrated in previous research [14]. German health insurance system is scattered in multiple companies, with some of them being more similar the others in terms of insurants and some known for more specific characteristics. Nevertheless, there is one system of hospitals where the patients are admitted. So, the insurance datasets are likely to differ in average hospitalization rates, but they should not generate disparate or differently weighted hospital networks.

For modelling HAI spread in the healthcare system, we used transmission parameters based on previous research [2, 3, 7, 8, 11] and varied those across a range of clinically relevant parameters. For example, an important factor in modelling multidrug-resistant pathogens acquired in healthcare institutions is that patients can become carriers without displaying symptoms of infection. Consequently, they might remain colonized for a long time, i.e. either undetected and, therefore, not treated or because some of the pathogens are overall difficult to remove, despite decolonization efforts [39]. Some estimates of clearance rate are available. However, clearance might not be homogeneous, and some patients can carry the pathogen much longer. If such patients are readmitted to the same hospital, or possibly to another hospital, for the same or unrelated reason, they can reintroduce the pathogen. This is particularly the case, if such patients receive antibiotic treatment, which can lead to selection of resistant strains and uncover a partially hidden colonization.

Prevalence estimates as a measure of epidemic spread in the network were robust until high level of incompleteness of 90% and more. Accounting for the fact that the datasets were themselves subsamples of the populations of the federal states, one could assume that 0.1 times 30–40%, i.e. a minimum coverage of 3–4% of the local population is required to represent the patient-movement network structure in the region relevant for pathogen transmission. In each region, typically several insurance companies have a larger share of 5% of coverage, although above 10% there usually only few, with AOKs insuring 30% or more of the local population in most federal states, followed by possibly other more local insurance companies and any of the three largest countrywide companies. In this sense for each federal state in Germany, there are 3–5 potential data sources for such analyses, which fulfil the requirement of the coverage of more than 10% of the local population. They would provide basis for robust modelling of pathogen spread in regional hospital networks. Conversely, for the whole of Germany there 1–2 health insurance companies which fulfil this criterion in most of the federal states, in addition to the composite of all local AOKs taken together.

### Limitations

We used data from only three insurance companies from selected regions, so that our findings might not be generalizable to other regions of Germany or to other countries. Also, there could be differences between insurance companies with respect to characteristics of the insurants, which could again affect generalization of our findings even within the studied regions. For example, a dataset from an insurance company with younger insurants who have less hospitalizations could tolerate lower fraction of the included population before too many transfer links are lost and epidemic model results are biased. In addition, we used only a relatively simple transmission model, and more complex infections could bring additional problems not addressed in our analyses. We assumed that all susceptible patients had the same probability of becoming infected within each hospital although it is known that the physical and organizational structures of a hospital affect these numbers [40, 41]. In our model, we also neglected the existence of smaller subunits as hospital wards. Patients are likely to transmit the pathogen to patients in the same ward and not to all patients in the hospital. Finally, we investigated only a range of assumptions regarding transmission parameters in the epidemiological model. While our focus was on parameters observed for MRSA and other multidrug-resistant pathogens transmitted in the hospitals, it could be that some specific pathogens were not covered in the chosen range or that new pathogens will emerge with different characteristics.

### Conclusions

Although hospital network measures were biased when incompleteness levels were high, networks based on incomplete data still maintained similar patient transfer patterns. In addition, even up to high levels of incompleteness, simulated infection prevalence in SIS transmission models displayed only small bias. At the upper boundary of incompleteness levels, scale-up improved the robustness of patient-movement based transmission model. While incompleteness of patient transfer data remains a challenge, our findings are reassuring that across a broad range of assumptions, robust estimates can be reached despite incomplete data.

## Supporting information

S1 Fig Distribution of number of hospitalizations per hospital in the three AOK datasets including hospitals with less than 100 hospitalizations during six-year time period. The x-axis represents hospitals, ranked from largest to smallest (left to right) based on the number of hospitalizations that occurred there. The dash lines indicate the bounds of 100 hospitalizations.
(EPS)

S2 Fig Density distribution of length of stay based on age and sex categories in AOK Bavaria. The upper and lower panels show the distributions for female and male patents, respectively.
(EPS)

S3 Fig Density distribution of length of stay based on age and sex categories in AOK Lower Saxony. The upper and lower panels show the distributions for female and male patents, respectively.
(EPS)

S4 Fig Density distribution of length of stay based on age and sex categories in AOK PLUS. The upper and lower panels show the distributions for female and male patents, respectively.
(EPS)

S5 Fig Distribution of number of hospitalizations per hospital in the three AOK datasets. The x-axis represents hospitals, ranked from largest to smallest (left to right) based on the number of hospitalizations that occurred there.
(EPS)

S6 Fig Distribution of weights for hospital edges. Here, the “Direct” and “Indirect” indicates the direct and indirect hospital networks, respectively.
(EPS)

S7 Fig Distribution of degrees on direct hospital networks. The x-axis represents hospitals, ranked from large to small (left to right) based on the number of hospitalizations that occurred there.
(EPS)

S8 Fig Distribution of degrees on indirect hospital networks. The x-axis represents hospitals, ranked from large to small (left to right) based on the number of hospitalizations that occurred there.
(EPS)

S9 Fig Distribution of strengths on direct hospital networks. The x-axis represents hospitals, ranked from large to small (left to right) based on the number of hospitalizations that occurred there.
(EPS)

S10 Fig Distribution of strengths on indirect hospital networks. The x-axis represents hospitals, ranked from large to small (left to right) based on the number of hospitalizations that occurred there.
(EPS)

S11 Fig Impact of sampling and scale-up on static direct hospital subnetwork measures in boxplots. We plotted the weighted absolute value of relative biases (weighted ARBs) across different network measures on sampled (inset) and scale-up (main plot) static hospital networks for various incompleteness levels of patient removed. The boxplots showed the progressive disparities of ARBs of diverse network measures as incompleteness increases. Each row specified the type of hospital subnetwork and the legend in the right side showed the corresponding network measures. Each column involved the graphs from the same AOK data.
(EPS)

S12 Fig Cosine similarities (CSs) in direction “In” between sampled and original hospital networks according to 9 incompleteness levels. The right y-axis displays incompleteness levels. The bar plots on the top of the heat maps indicate hospital sizes, ranking from highest to lowest (left to right) based on the number of hospitalizations that occurred there, which also defines the x-axis.
(EPS)

S13 Fig Cosine similarities (CSs) in direction “Out” between sampled and original hospital networks according to 9 incompleteness levels. The right y-axis displays incompleteness levels. The bar plots on the top of the heat maps indicate hospital sizes, ranking from highest to lowest (left to right) based on the number of hospitalizations that occurred there, which also defines the x-axis.
(EPS)

S14 Fig Cosine similarities (CSs) in direction “In” between scale-up and original hospital networks according to 9 incompleteness levels. The right y-axis displays incompleteness levels. The bar plots on the top of the heat maps indicate hospital sizes, ranking from highest to lowest (left to right) based on the number of hospitalizations that occurred there, which also defines the x-axis.
(EPS)

S15 Fig Cosine similarities (CSs) in direction “Out” between scale-up and original hospital networks according to 9 incompleteness levels. The right y-axis displays incompleteness levels. The bar plots on the top of the heat maps indicate hospital sizes, ranking from highest to lowest (left to right) based on the number of hospitalizations that occurred there, which also defines the x-axis.
(EPS)

S16 Fig Daily prevalence PRBs in AOK Bavaria according to incompleteness levels equal or above 90%. The red and dark blue dashed lines indicate the bounds of 5% and 10% estimation biases.
(EPS)

S17 Fig Daily prevalence PRBs in AOK Lower Saxony according to incompleteness levels equal or above 90%. The red and dark blue dashed lines indicate the bounds of 5% and 10% estimation biases.
(EPS)

S18 Fig Daily prevalence PRBs in AOK PLUS according to incompleteness levels equal or above 90%. The red and dark blue dashed lines indicate the bounds of 5% and 10% estimation biases.
(EPS)

S19 Fig Prevalence in hospital nodes obtained from transmission model simulations for different AOK datasets. The dashed lines in each graph point towards the x-axis of prevalence peaks.
(EPS)

S20 Fig Final prevalence relative biases (PRBs) in community nodes for varying transmission parameters. The final PRBs in community nodes were calculated based on the simulations on AOK PLUS patient-movement networks, built by scale-up at incompleteness levels of 96% and 98%. In (a) we presented the final PRBs in community nodes for different values of *β*. In (b) we presented the final PRBs in community nodes for different values of *γ*.
(EPS)
